# Dose and normal tissue complication probability analysis of various radiotherapy regimens for thymomas

**DOI:** 10.3389/fonc.2026.1730138

**Published:** 2026-02-05

**Authors:** Wu Xiandong, Luo Haifeng, Zhichao Wang, Li Chao, Hu Yan, Li Jingjing

**Affiliations:** Department of Oncology, First People’s Hospital of Jingzhou, Jingzhou, China

**Keywords:** non-coplanar VMAT, NTCP, radiation dose, radiation therapy, thymic tumor

## Abstract

**Objective:**

This study aimed to compare dosimetric differences and normal tissue complication probability (NTCP) among coplanar intensity-modulated radiation therapy (CO-IMRT), non-coplanar IMRT (NONCO-IMRT), coplanar volumetric-modulated arc therapy (CO-VMAT), and non-coplanar VMAT (NONCO-VMAT) in thymoma radiotherapy and clarify the value of non-coplanar plans.

**Methods:**

Forty-eight post-thymoma surgery patients were enrolled in this study. Four radiotherapy plans (50 Gy/25 fractions) were made for each patient. The target conformity index (CI), homogeneity index (HI), and organ-at-risk (OAR) (lungs, heart, etc.) dose parameters were then evaluated. NTCP was calculated using the Lyman–Kutcher–Burman (LKB) model. Statistical analysis was performed using the Student–Newman–Keuls (SNK-q) test (p < 0.05 for significance).

**Results:**

There were no significant clinical differences in target volume coverage and homogeneity among the four plans. The lung V_20_, heart V_30_, spinal cord D_max_, and esophagus D_max_ of the VMAT techniques were all lower than those of IMRT (p < 0.001). The breast dose of CO-VMAT was significantly higher, while the breast parameters of NONCO-VMAT were close to those of IMRT. NTCP results were consistent with dosimetric findings: VMAT plans had lower NTCP for the heart, spinal cord, and esophagus; moreover, the breast NTCP of CO-VMAT was significantly increased (1.75%), while that of NONCO-VMAT decreased to 0.28%, which was similar to that of IMRT.

**Conclusion:**

All four plans meet the thymoma target dose requirement. However, NONCO-VMAT is preferred for its advantages in multi-organ protection and balanced breast dose. Additionally, CO-VMAT should be used cautiously in female patients due to the higher breast dose.

## 1 Introduction

Thymomas and thymic carcinomas are rare mediastinal tumors that arise from thymic epithelial cells and account for 0.2%–1.5% of all malignancies (1, 2). Thymic carcinoma is more aggressive, accounting for approximately 20% of thymic epithelial tumors (3, 4). Surgery remains the primary treatment for thymoma; however, radiotherapy (RT) also plays an important role, particularly in adjuvant and palliative settings (5), due to the intrinsic radiosensitivity of these tumors. Adjuvant RT has been shown to improve overall survival (OS) in patients with Masaoka–Koga stage IIB disease or those with aggressive histologic subtypes following resection (6, 7). In cases where complete surgical resection is not feasible, postoperative RT may delay or prevent recurrence and prolong patients’ survival (8, 9). For thymic carcinoma, postoperative RT is often recommended even after complete resection, with RT being optional for stage I, advised for stage II, and strongly recommended for stage III/IV disease (10).

Minimizing radiation exposure to surrounding normal tissues is a major challenge in thymoma radiotherapy. Due to its location in the anterior mediastinum, conventional radiation plans typically adopt anterior beam arrangements, resulting in unavoidable exposure of adjacent critical organs such as the lungs, heart, and esophagus to radiation (11, 12). Among these, the lungs and heart are particularly susceptible to radiation-induced toxicity, with previous studies such as those by Jalbout et al. identifying the lungs as the organ at the greatest risk (13).

In recent years, several strategies have been explored to mitigate radiation-related toxicity in thymoma treatment. Techniques such as intensity-modulated proton therapy (IMPT) (14–17) and deep inspiration breath-hold (DIBH) (18) have demonstrated efficacy in reducing radiation doses to intrathoracic organs, thereby lowering the risk of late complications and secondary malignancies. However, widespread adoption of these approaches is limited by the high cost of proton facilities and the technical demands of DIBH, which requires patient compliance and dedicated respiratory-gating systems. In numerous resource-limited centers, these technologies are not readily accessible.

As an alternative, modifying beam geometry, specifically through non-coplanar radiotherapy techniques, offers a more practical means of reducing organ-at-risk (OAR) doses (19). Non-coplanar intensity-modulated radiation therapy (NONCO-IMRT) employs radiation beams delivered from multiple non-parallel planes, improving dose conformity by reducing the overlap of entrance and exit doses through critical structures (20). Dong et al. reported that in stereotactic body radiotherapy (SBRT) for lung tumors, NONCO-IMRT allowed for a 20-Gy increase in tumor dose compared to volumetric-modulated arc therapy (VMAT) while still respecting standard OAR dose constraints (21). Some studies have also suggested that non-coplanar planning may increase lung V_5_ but reduce lung V_10_ and V_20_, potentially offering better sparing of lung tissue compared to conventional coplanar IMRT (CO-IMRT) (22).

However, studies on non-coplanar VMAT (NONCO-VMAT) in thymomas are relatively scarce. This study aimed to explore the dosimetric differences between non-coplanar plans in IMRT and VMAT while analyzing the normal tissue complication probability (NTCP) of OARs in different plans. The aim of this study was to clarify the advantages and disadvantages of non-coplanar plans in radiation therapy for thymoma.

## 2 Methods

### 2.1 Patient selection and data collection

All procedures were conducted in accordance with relevant guidelines and institutional regulations. Overall, 48 patients diagnosed with thymoma or thymic carcinoma who received postoperative radiotherapy between 2019 and 2025 were retrospectively included in this study. The cohort comprised 24 male and 24 female patients, with a median age of 53 years (range, 27–70 years). Among them, 30 patients were diagnosed with thymomas, including 23 with Masaoka–Koga stage II and seven with stage III disease. The remaining 18 patients had thymic carcinoma, of whom nine were stage II, seven were stage III, and two were stage IV. All radiotherapy regimens for the enrolled patients were determined after evaluation by the multidisciplinary team for thymic tumors at our hospital.

This study was designed as a retrospective study and aimed to compare the dosimetric differences among various radiotherapy techniques. The study did not involve direct contact with the patients and was a non-interventional retrospective study. Therefore, it was approved by the Medical Ethics Committee of Jingzhou First People's Hospital (KY202405) and the need for obtaining informed consent from the participants was waived. The study was performed in accordance with the ethical standards as laid down in the 1964 Declaration of Helsinki.

All patients underwent CT simulation with a slice thickness of 5 mm, and the images were transferred to the uRT-TPOIS treatment planning system for further planning.

### 2.2 Target volumes and OAR delineation

The gross tumor volume (GTV) was defined as the visible tumor bed or residual lesion on postoperative imaging. The clinical target volume (CTV) was generated by expanding the GTV by 0.5 cm to include areas of potential microscopic disease, such as adjacent thymic tissues or potentially involved structures. The planning target volume (PTV) was created by further expanding the CTV by 0.5 cm to account for setup and motion uncertainties. OARs delineated included the bilateral lungs, heart, esophagus, and spinal cord.

### 2.3 Treatment planning and beam configuration

Treatment plans were generated using the uRT-TPOIS system (United Imaging, Shanghai, China) and delivered on a uRT linac 506c linear accelerator (United Imaging, Shanghai, China). All plans utilized 6-MV photon beams, and dose calculations were performed using the Monte Carlo method. Forty-six patients received adjuvant radiotherapy, with no residual lesions. The prescribed dose was 50 Gy in 25 fractions. The remaining two patients with stage IV thymic carcinomas were initially planned to first receive 50 Gy in 25 fractions, with an intended subsequent cone-down boost to 66 Gy based on tumor response. However, after completing 50 Gy, radiation pneumonitis was observed on follow-up imaging, and further boosts were discontinued. These two cases were still included in the present dosimetric comparison, as they received the initial 50 Gy course.

Each patient underwent four treatment planning approaches: CO-IMRT, NONCO-IMRT, coplanar VMAT (CO-VMAT), and NONCO-VMAT. For all plans, the dose to 95% of the PTV (V95%) was required to cover at least 99% of the prescribed dose. Additionally, dose distributions to the lungs, heart, and spinal cord were evaluated for comparison. The dose constraint parameters for organs at risk are shown in **Table 1**.

**Table 1:** Dose constraints used for the generation of treatment plans for thymoma.

OAR	Dose constraint
Lung	V_5_ < 50%
	V20 < 20%
	Vmean < 12 Gy
Heart	V30 < 40%
	D_mean_ < 26 Gy
Spinal cord	D_max_ < 40 Gy
Breast	V_mean_ < 5 Gy
Esophagus	D_max_ < 50 Gy

D_mean_, average dose; V_i_Gy, organ volume receiving more than iGy; D_max_, maximum dose; OAR, organ at risk.

CO-IMRT included five beam angles—330°, 345°, 0°, 15°, and 30°—all with a couch angle of 0°. For NONCO-IMRT, three fields (330°, 0°, and 30°) remained coplanar (couch angle 0°), while the other two fields used the same gantry angles (330° and 30°) but were delivered with a couch angle of 270°. CO-VMAT included a double arc, an angle of 275°–85°, and a couch angle of 0°. In NONCO-VMAT, two arc angles were 300°–60° with a bed angle of 0, two arc angles of 330°–30°, and a couch angle of 90°. All modalities involved avoiding mechanical collisions during delivery.

### 2.4 Dosimetric evaluation and indices

Dosimetric assessment included both target conformity and dose to OARs. The conformity index (CI) was calculated as the ratio of the PTV covered by the 95% isodose line to the total PTV. A CI value close to 1 indicates optimal conformity. In this study, all plans achieved a CI exceeding 0.99. $Heterogeneity\;index(HI)=\frac{D_{2\%}-D_{98\%}}{D_{50\%}}$. *D*_2%_ and *D*_98%_ represent the high and low dose absorption regions of PTV, respectively (23). A lower HI value indicates a more uniform dose distribution within the target area.

For OARs, the following parameters were analyzed: whole lung V_20_, V_5_, and D_mean_; heart V_30_ and D_mean_; breast D_mean_; esophagus D_max_; and spinal cord D_max_.

### 2.5 Radiobiological parameters

The Lyman–Kutcher–Burman (LKB) NTCP model describes the NTCP as an organ or tumor sub-volume function that receives a uniform dose (24) of radiation. Because the dose distribution is heterogeneous in clinical treatment, a dose–volume histogram (DVH)-reduction method with the concept of Equivalent Uniform Dose (EUD) is typically employed (25). EUD represents a single uniform dose value equivalent to the biological effect of the non-uniform dose distribution delivered to an organ or tumor.


NTCP=1/2π∫−∞te−x22dx,t=(EUD−D50)/mD50,EUD=(∑iviDi1n)n

where *v_i_* is the relative volume receiving the corresponding *i*th dose bin with the dose *D_i_* of a differential DVH. The parameter *D*_50_ is the dose that corresponds to a 50% probability of complication, *m* is the slope of the NTCP curve, and *n* represents the volume dependency of the risk of complications (26). The parameters employed for calculating the NTCP of the lungs, heart, spinal cord, breast, and esophagus are shown in **Table 2** (27–30).

**Table 2:** Parameters used for the calculation of normal tissue complication probability.

OAR	n	m	D_50_	Endpoint
Lung	1	0.37	30.5	Pneumonitis
Heart	0.78	0.59	43.3	Major adverse cardiac events
Spinal cord	0.05	0.175	66.5	Myelopathy
Esophagus	0.29	0.15	46	Grade 2 esophagitis
Breast	0.36	0.19	60	Fibrosis

Major adverse cardiac events were defined as acute myocardial infarction [International Classification of Diseases, 10th Revision (ICD-10) I21–I24], unstable angina (ICD-10, I20), coronary revascularization, coronary artery bypass grafting surgery, heart failure with hospitalization, or death from cardiac disease.

### 2.6 Statistical analysis

The statistical analysis was performed using the SPSS version 17.0 software. Data were represented as (x ± s). Paired comparisons between different plans were conducted using the Student–Newman–Keuls (SNK-q) test. A two-sided p-value <0.05 was considered statistically significant.

## 3 Results

### 3.1 Planning dose parameters

**Table 3** summarizes the dosimetric parameters, including the dose of CI and HI of the PTV, as well as a comparison with the doses of normal organs. **Table 4** presents the statistical comparison of the target volumes and OAR doses among the four radiotherapy techniques for thymomas.

**Table 3:** Comparison of dose distribution for OARs between four plans (x ± s, n = 48).

OAR	Parameter	CO-IMRT	NON-IMRT	CO-VMAT	NON-VMAT
PTV	CI	0.997 ± 0.004	0.999 ± 0.002	0.999 ± 0.002	0.999 ± 0.002
	HI	0.090 ± 0.017	0.081 ± 0.008	0.081 ± 0.014	0.083 ± 0.011
Lung	V_5_	30.742 ± 10.790	31.999 ± 11.583	31.790 ± 9.89	31.328 ± 10.732
	V_20_	11.694 ± 6.490	11.018 ± 6.852	9.465 ± 5.252	9.461 ± 5.627
	V_mean_	6.878 ± 2.819	6.93 ± 3.029	6.769 ± 2.377	6.694 ± 2.501
Heart	V_30_	11.550 ± 7.018	11.162 ± 6.693	7.897 ± 3.793	8.408 ± 4.304
	V_mean_	7.844 ± 3.942	8.305 ± 3.932	6.664 ± 2.642	7.041 ± 2.849
Spinal cord	D_max_	28.909 ± 9.716	28.23 ± 9.764	24.073 ± 7.583	24.316 ± 7.556
Breast	D_mean_	3.372 ± 2.022	3.162 ± 2.131	6.786 ± 3.559	3.587 ± 1.840
	V_5_	9.287 ± 4.998	10.361 ± 5.591	35.888 ± 16.120	16.690 ± 7.400
	V_10_	8.321 ± 4.657	8.823 ± 5.126	24.729 ± 13.312	12.295 ± 6.097
Esophagus	D_max_	39.990 ± 11.578	39.976 ± 11.653	37.246 ± 13.388	38.246 ± 12.731
	D_mean_	12.498 ± 5.960	13.207 ± 5.967	10.733 ± 5.850	11.114 ± 5.554

OARs, organs at risk; IMRT, intensity-modulated radiation therapy; CO-IMRT, coplanar IMRT; NONCO-IMRT, non-coplanar IMRT; VMAT, volumetric-modulated arc therapy; CO-VMAT, coplanar VMAT; NONCO-VMAT, non-coplanar VMAT; PTV, planning target volume; CI, conformity index; HI, homogeneity index.

**Table 4:** Statistical comparison of target volume and OAR doses among the four radiotherapy techniques for thymoma.

		p-Value					
OAR	Parameter	CO-IMRT *vs*. NON-IMRT	CO-IMRT *vs*. CO-VMAT	CO-IMRT *vs*. NON-VMAT	NON-IMRT *vs*. CO-VMAT	NON-IMRT *vs*. NON-VMAT	CO-VMAT *vs*. NON-VMAT
PTV	CI	0.006	0.066	0.173	0.298	0.167	<0.001
	HI	0.187	0.038	0.093	0.427	0.023	<0.001
Lung	V_5_	<0.001	<0.001	<0.001	<0.001	<0.001	<0.001
	V_20_	<0.001	<0.001	<0.001	<0.001	<0.001	<0.001
	V_mean_	<0.001	<0.001	<0.001	<0.001	<0.001	<0.001
Heart	V_30_	<0.001	<0.001	<0.001	<0.001	<0.001	<0.001
	V_mean_	<0.001	<0.001	<0.001	<0.001	<0.001	<0.001
Spinal cord	D_max_	<0.001	<0.001	<0.001	<0.001	<0.001	<0.001
Breast	D_mean_	<0.001	<0.001	<0.001	<0.001	<0.001	<0.001
	V_5_	<0.001	<0.001	<0.001	<0.001	<0.001	<0.001
	V_10_	<0.001	0.902	<0.001	0.911	<0.001	0.752
Esophagus	D_max_	<0.001	<0.001	<0.001	<0.001	<0.001	<0.001
	D_mean_	<0.001	<0.001	<0.001	<0.001	<0.001	<0.001

OAR, organ at risk; IMRT, intensity-modulated radiation therapy; CO-IMRT, coplanar IMRT; NONCO-IMRT, non-coplanar IMRT; VMAT, volumetric-modulated arc therapy; CO-VMAT, coplanar VMAT; NONCO-VMAT, non-coplanar VMAT; PTV, planning target volume; CI, conformity index; HI, homogeneity index.

The differences in PTV dose parameters among the four plans were minimal, with statistical significance only observed between CO-VMAT and NONCO-VMAT. Regarding the breast V_10_, no statistical significance was found in the comparisons of CO-IMRT *vs*. CO-VMAT and CO-VMAT *vs*. NONCO-VMAT. For other comparisons between plans, the doses to OARs showed significant statistical differences (p < 0.001). The lung V_20_ values of CO-VMAT and NONCO-VMAT (9.465 and 9.461, respectively) were significantly lower than those of IMRT techniques (11.694 for CO-IMRT and 11.018 for NONCO-IMRT). The differences in lung V_5_ and lung V_mean_ among the four techniques were also small. VMAT showed a slight advantage in lung V_mean_, with the lowest value of 6.694 observed in NONCO-VMAT. IMRT showed a slight advantage in lung V_5_, with the lowest value of 30.742 observed in CO-IMRT.

The heart V_30_ values of CO-VMAT (7.897) and NONCO-VMAT (8.408) were significantly lower than those of IMRT techniques (11.550 for CO-IMRT and 11.162 for NONCO-IMRT). The heart D_mean_ of VMAT plans was also lower than that of IMRT plans. Among them, CO-VMAT (6.664) had the lowest heart D_mean_, outperforming other techniques.

The spinal cord D_max_ of VMAT techniques (24.073 for CO-VMAT and 24.316 for NONCO-VMAT) was lower than that of IMRT techniques (28.909 for CO-IMRT and 28.23 for NONCO-IMRT). Regarding the esophagus D_max_, VMAT techniques (37.246 for CO-VMAT and 38.246 for NONCO-VMAT) also showed lower values compared to IMRT techniques (≈39.99). Additionally, the esophagus D_mean_ of VMAT plans was lower, with CO-VMAT achieving the lowest value (10.733).

The breast D_mean_ (6.786), V_5_ (35.888), and V_10_ (24.729) of CO-VMAT were significantly higher than those of other techniques. The breast parameters of NONCO-VMAT (D_mean_ of 3.587, V_5_ of 16.690, and V_10_ of 12.295) were superior to those of CO-VMAT and even close to those of IMRT techniques.

**Figure 1:**
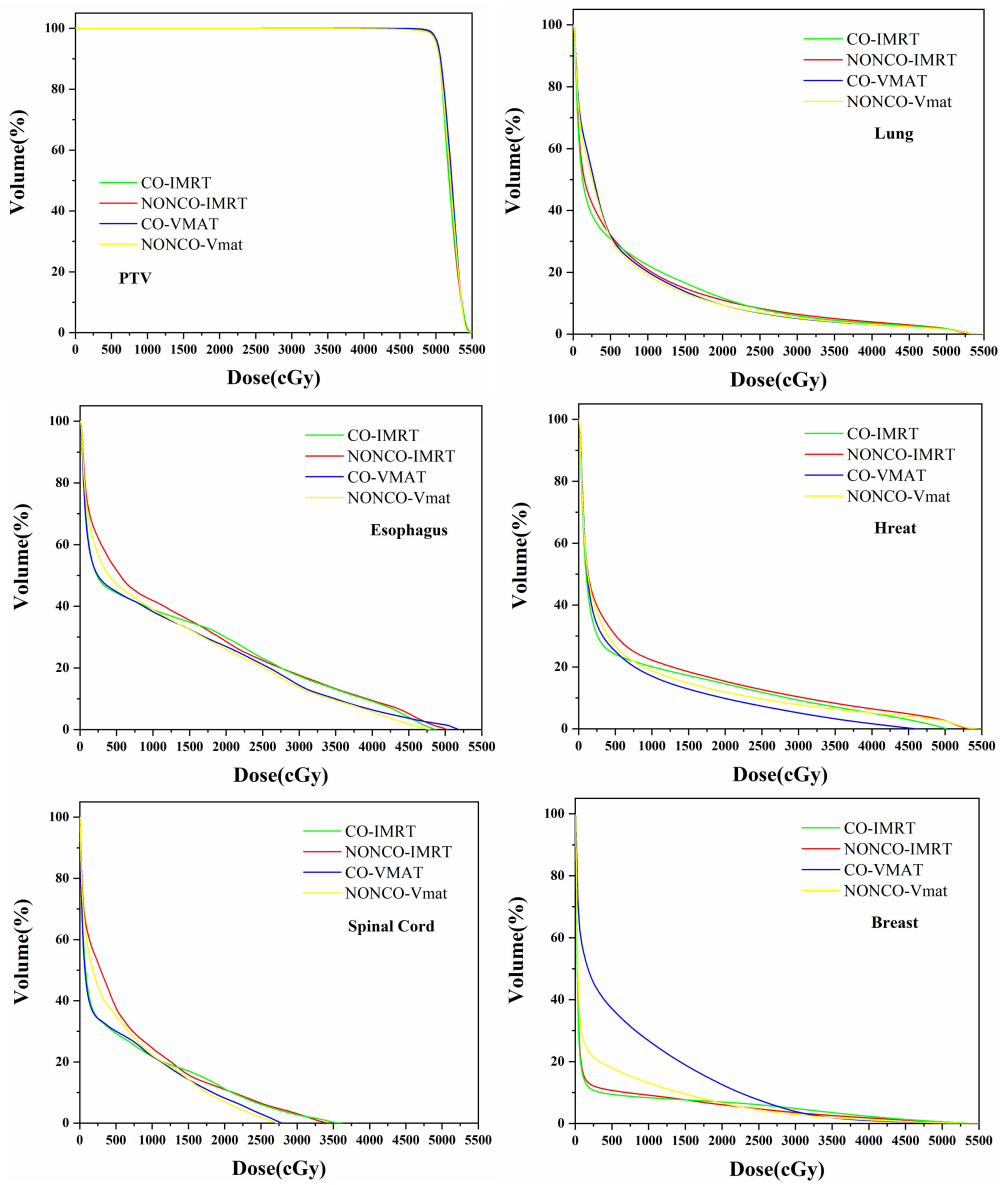
DVH average of 48 patients for PTV and critical organs in CO-IMRT, NONCO-IMRT, CO-VMAT, and NONCO-VMAT plans. DVH, dose–volume histogram; PTV, planning target volume; IMRT, intensity-modulated radiation therapy; CO-IMRT, coplanar IMRT; NONCO-IMRT, non-coplanar IMRT; VMAT, volumetric-modulated arc therapy; CO-VMAT, coplanar VMAT; NONCO-VMAT, non-coplanar VMAT.

**Figure 2:**
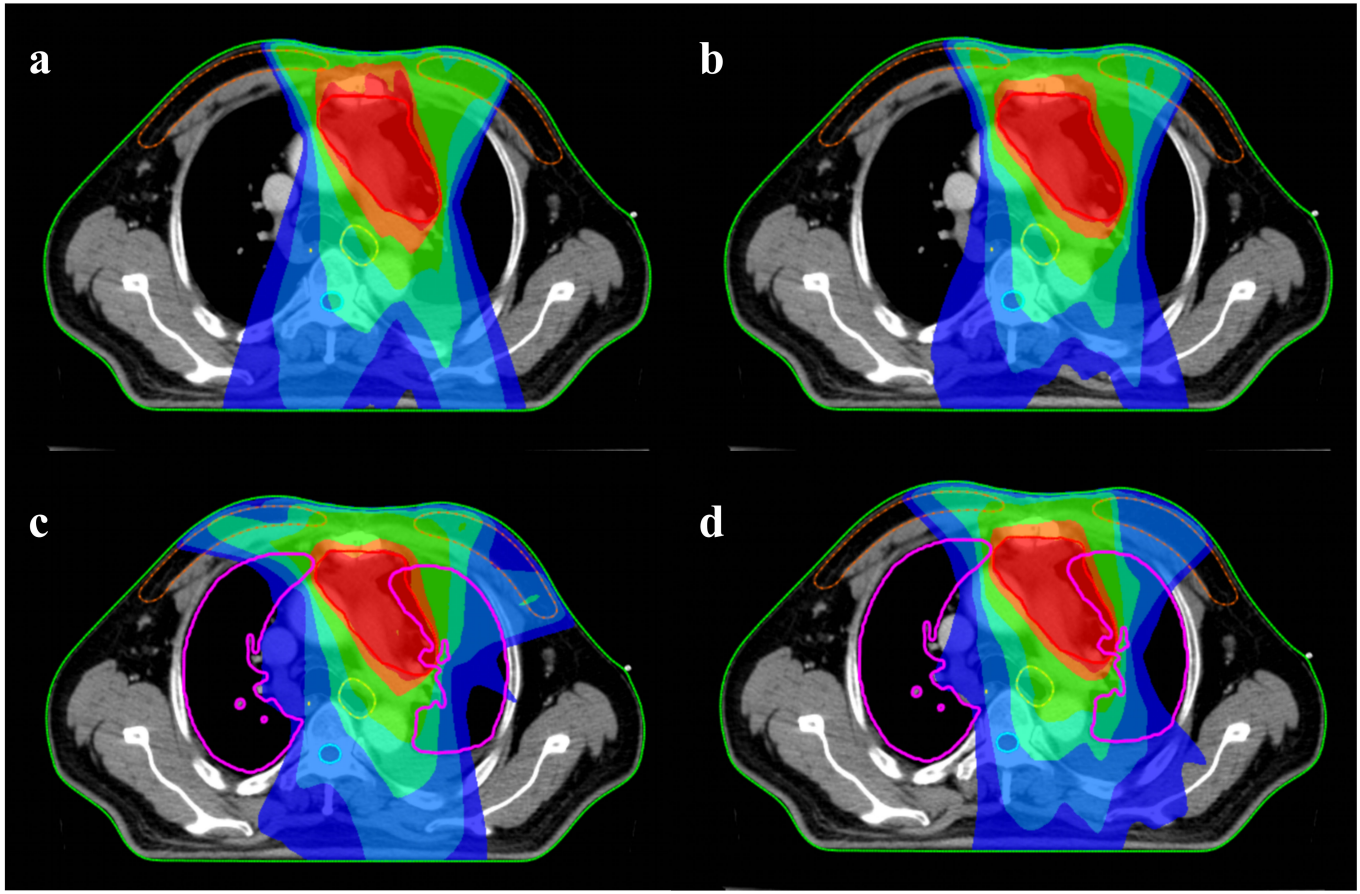
Typical dose distributions around the target volume when female thymoma patients receive different radiotherapy techniques: **(a)** CO-IMRT, **(b)** NONCO-IMRT, **(c)** CO-VMAT, and **(d)** NONCO-VMAT. The red, orange, green, yellow, light blue, and dark blue colors represent the isodose lines of 50, 45, 30, 20, 10, and 5 Gy, respectively. IMRT, intensity-modulated radiation therapy; CO-IMRT, coplanar IMRT; NONCO-IMRT, non-coplanar IMRT; VMAT, volumetric-modulated arc therapy; CO-VMAT, coplanar VMAT; NONCO-VMAT, non-coplanar VMAT.

**Figure 1** shows the mean DVHs of the PTV and the mean DVH of key organs for patients in the CO-IMRT, NONCO-IMRT, CO-VMAT, and NONCO-VMAT. **Figure 2** presents the typical dose distributions around the target volume when female thymoma patients receive different radiotherapy techniques.

### 3.2 Radiobiological parameters

Using the mean DVH of the four plans, the NTCP of OARs for the four different radiotherapy techniques was calculated, as shown in **Table 5**. Among the four plans, the lung NTCP of CO-VMAT was the lowest (1.58%); however, the overall difference was small (ranging from 1.58% to 1.66%). The heart NTCP of VMAT plans (7.74% for CO-VMAT and 8.84% for NONCO-VMAT) was lower than that of IMRT plans (8.96% for CO-IMRT and 9.77% for NONCO-IMRT). The spinal cord NTCP of VMAT plans (0.0079% for CO-VMAT and 0.0062% for NONCO-VMAT) was significantly lower than that of IMRT plans (0.049% for CO-IMRT and 0.038% for NONCO-IMRT); however, all values were far below the clinical warning threshold. The esophagus NTCP of VMAT plans (0.10% for CO-VMAT and 0.06% for NONCO-VMAT) was lower than that of IMRT plans (0.16% for CO-IMRT and 0.19% for NONCO-IMRT). The breast NTCP of CO-VMAT (1.75%) was significantly higher than that of other plans. The breast NTCP of CO-IMRT (0.01%) was much lower than that of other plans, while the breast NTCPs of NONCO-IMRT (0.23%) and NONCO-VMAT (0.28%) were similar.

**Table 5:** Normal tissue complication probability (NTCP) from average DVH for the four plans.

	NTCP (%)				
Technique	Lung	Heart	Spinal cord	Esophagus	Breast
CO-IMRT	1.60	8.96	0.049	0.16	0.010
NONCO-IMRT	1.66	9.77	0.038	0.19	0.23
CO-VMAT	1.64	7.74	0.0079	0.10	1.75
NONCO-VMAT	1.58	8.84	0.0062	0.060	0.28

DVH, dose–volume histogram; IMRT, intensity-modulated radiation therapy; CO-IMRT, coplanar IMRT; NONCO-IMRT, non-coplanar IMRT; VMAT, volumetric-modulated arc therapy; CO-VMAT, coplanar VMAT; NONCO-VMAT, non-coplanar VMAT.

## 4 Discussion

This study compared four radiotherapy planning schemes for the treatment of thymomas, including CO-IMRT, NONCO-IMRT, CO-VMAT, and NONCO-VMAT, for administering the same prescribed dose of 50 Gy/25 f. All plans were formulated with priority given to meeting the prescribed dose. The optimization criteria for OARs were consistent across the four plans for the same patient.

All four plans achieved excellent conformity (CI > 0.99) and satisfactory uniformity (HI 0.081–0.090) for the PTV, indicating that each technique can effectively deliver the prescribed dose to the target. This aligns with the primary goal of radiotherapy, ensuring adequate tumor control, while confirming that both IMRT and VMAT, whether coplanar or non-coplanar, are capable of meeting clinical target dose requirements for thymomas.

The most striking findings lie in the differential sparing of OARs across techniques, with VMAT (both CO-VMAT and NONCO-VMAT) generally outperforming IMRT in protecting critical structures, including the heart, spinal cord, and esophagus, while showing mixed results for the lungs and breasts.

VMAT techniques (CO-VMAT and NONCO-VMAT) significantly reduced lung V_20_ compared to IMRT techniques (9.46 *vs*. 11.02–11.69), a parameter strongly associated with radiation-induced pneumonitis risk (13, 26). Although lung V_5_ and V_mean_ showed smaller differences, the reduction in V_20_ was clinically meaningful, as V_20_ > 30% is a well-established predictor of pneumonitis. This aligns with the findings of previous studies suggesting that arc-based techniques (VMAT) can reduce high-dose lung exposure by optimizing beam angles to avoid overlapping with lung tissue (21).

VMAT plans (especially CO-VMAT) achieved lower heart V_30_ and D_mean_ than IMRT, with CO-VMAT reducing V_30_ by 30% (7.90 *vs*. 11.16–11.55). Given that heart V_30_ is linked to major adverse cardiac events (MACEs) (27), this reduction could translate to lower long-term cardiac toxicity, a critical consideration for patients with thymomas who often have prolonged survival.

VMAT techniques consistently reduced maximum doses to the spinal cord (24.07–24.32 *vs*. 28.23–28.91 Gy) and esophagus (37.25–38.25 *vs*. ~39.99 Gy) compared to IMRT. These findings support the utility of VMAT in sparing posterior (spinal cord) and central (esophagus) structures; moreover, more irradiation angles of VMAT technology make the medium- and high-dose curves wrap more closely.

A key exception was the breast, where CO-VMAT showed significantly higher D_mean_, V_5_, and V_10_ compared to other techniques. This likely stems from the arc angles used in CO-VMAT (275°–85° with 0°couch), which may irradiate more breast tissue in anterior mediastinal targets, particularly in female patients. **Figure 2** shows that the 5-Gy dose range of the CO-VMAT program covers more of the breast. In contrast, NONCO-VMAT (incorporating 90°couch angles) mitigated this effect, with breast parameters comparable to those of IMRT, highlighting the value of non-coplanar beam geometry in reducing unnecessary breast exposure, which is an important consideration for younger female patients at risk of breast fibrosis (29).

To assess the specific impact of dose differences on patients’ side effects, it was also necessary to combine the measurement of radiobiological indices. This study calculated the NTCP caused by thymoma radiotherapy to the lungs, heart, spinal cord, esophagus, and breasts, which correspond to pneumonia, major adverse cardiac events, myelopathy, grade 2 esophagitis, and fibrosis, respectively. The NTCP results were consistent with the dosimetric results. The VMAT plans also showed a lower complication risk for most OARs. Lung NTCP was the lowest in NONCO-VMAT (1.58%), consistent with its favorable V_20_. Heart NTCP was reduced in VMAT (7.74%–8.84% *vs*. 8.96%–9.77% in IMRT), aligning with lower V_30_ and D_mean_. Spinal cord NTCP was negligible across all plans but significantly lower in VMAT (0.0062%–0.0079% *vs*. 0.038%–0.049% in IMRT), confirming effective sparing. Esophagus NTCP was the lowest in NONCO-VMAT (0.06%), reflecting reduced D_max_ and D_mean_. The elevated breast NTCP in CO-VMAT (1.75%), compared to values of 0.01%–0.28% in other plans, further underscores the need to avoid excessive breast irradiation in clinical practice, particularly for female patients.

Our findings build on prior research on non-coplanar techniques and VMAT in thoracic malignancies. Dong et al. reported that non-coplanar IMRT (NONCO-IMRT) improved the protection of OARs in lung SBRT (21). This study extends this application to thymomas, showing that NONCO-VMAT is superior to CO-VMAT in balancing target volume coverage and breast protection. Furthermore, the advantage of VMAT in reducing the doses to the heart and spinal cord was consistent with the findings of studies on lung cancer and esophageal cancer, which reported that arc-based techniques can minimize the radiation dose received by adjacent structures (19, 22). He et al. (31) found that CO-VMAT plans increased the radiation dose to the breasts, which is consistent with the results of our study. However, our study further demonstrates that NONCO-VMAT can significantly reduce the breast dose in VMAT plans while maintaining other dosimetric advantages of VMAT plans.

This study has several limitations. The study’s retrospective design and small cohort of only 48 patients may introduce selection bias; therefore, larger prospective cohorts are needed to validate clinical outcomes (e.g., pneumonitis and cardiac events). NTCP calculations relied on the Lyman model with parameters from other tumor types (26–29); therefore, thymoma-specific NTCP parameters were lacking and warrant further investigation. The findings of this study inspire us to explore new techniques that could be extended to patients with locally advanced, non-surgical thymoma or thymic carcinoma. In such cases, the target volumes are typically larger and more irregular in shape and may have more complex relationships with adjacent OARs. Evaluating whether different radiotherapy techniques, particularly non-coplanar VMAT, can retain their superior protective capacity for OARs while ensuring target dose coverage in these more complex clinical scenarios would be of significant clinical value.

## 5 Conclusion

In summary, all four radiotherapy techniques achieved adequate target coverage for thymomas; however, VMAT, particularly NONCO-VMAT, offered superior OAR sparing for the heart, spinal cord, esophagus, and lungs, with balanced breast protection. These findings support the use of NONCO-VMAT as a treatment option for most patients with thymoma, especially those requiring heart and spinal cord sparing, while CO-VMAT may be avoided in female patients due to the associated higher breast dose.

## Data Availability

The data analyzed in this study is subject to the following licenses/restrictions: Limitations on data set: The data set used in this retrospective study is not publicly available due to patient privacy and ethical restrictions. It consists of historical planning CT images from a single institutional radiotherapy planning system. Access is restricted under the approval of the Institutional Review Board (IRB) of our hospital. Anonymized data may be available from the corresponding author upon reasonable request and with permission from the IRB. Requests to access these datasets should be directed to WX, 127004938@qq.com.
